# *Staphylococcus aureus* Protein A Binds to Osteoblasts and Triggers Signals That Weaken Bone in Osteomyelitis

**DOI:** 10.1371/journal.pone.0018748

**Published:** 2011-04-15

**Authors:** Tania Claro, Amro Widaa, Maghnus O'Seaghdha, Helen Miajlovic, Timothy J. Foster, Fergal J. O'Brien, Steven W. Kerrigan

**Affiliations:** 1 Molecular and Cellular Therapeutics, Royal College of Surgeons in Ireland, Dublin, Ireland; 2 Moyne Institute of Preventive Medicine, Trinity College Dublin, Dublin, Ireland; 3 Department of Anatomy, Royal College of Surgeons in Ireland, Dublin, Ireland; 4 Trinity Centre for Bioengineering, Trinity College Dublin, Dublin, Ireland; 5 School of Pharmacy, Royal College of Surgeons in Ireland, Dublin, Ireland; University of Edinburgh, United Kingdom

## Abstract

Osteomyelitis is a debilitating infectious disease of the bone. It is predominantly caused by *S. aureus* and is associated with significant morbidity and mortality. It is characterised by weakened bones associated with progressive bone loss. Currently the mechanism through which either bone loss or bone destruction occurs in osteomyelitis patients is poorly understood. We describe here for the first time that the major virulence factor of *S. aureus,* protein A (SpA) binds directly to osteoblasts. This interaction prevents proliferation, induces apoptosis and inhibits mineralisation of cultured osteoblasts. Infected osteoblasts also increase the expression of RANKL, a key protein involved in initiating bone resorption. None of these effects was seen in a mutant of *S. aureus* lacking SpA. Complementing the SpA-defective mutant with a plasmid expressing *spa* or using purified protein A resulted in attachment to osteoblasts, inhibited proliferation and induced apoptosis to a similar extent as wildtype *S. aureus*. These events demonstrate mechanisms through which loss of bone formation and bone weakening may occur in osteomyelitis patients. This new information may pave the way for the development of new and improved therapeutic agents to treat this disease.

## Introduction

The skeleton is a dynamic organ system which is constantly being rejuvenated and remodelled [1]. Bone remodelling involves the coordinated effort of osteoclasts and osteoblasts. Osteoclasts drive resorption of bone by acidification and release of lysosomal enzymes. Osteoblasts are responsible for the deposition of bone matrix and are thought to facilitate the calcification and mineralisation of the bone matrix. Together these cells function to ensure healthy bone, giving strength and rigidity in the skeletal system [2]. When pathogenic bacteria gain entry to healthy bone this leads to a condition called osteomyelitis.

Osteomyelitis is a debilitating infectious disease of the bone which is associated with significant morbidity and mortality [3]. It typically develops in the distal femur and proximal tibia in children and in adults, but any bone can be infected [4]. It is characterised by severe inflammation and progressive bone destruction [5]. Infection can occur as a result of haematogeneous spread or from a contiguous source secondary to an open injury to bone caused by trauma, bone surgery or joint replacement [6]. As part of the infectious process dead or dying bone can detach from healthy bone forming an island surrounded by a ring of sclerosis, which in turn leads to the development of an independent focus of infection [7]. In acute and chronic disease there is some degree of uncontrolled bone remodelling which often leads to an associated deformity [8]. Treatment of osteomyelitis involves aggressive antibiotic therapy which is often coupled with surgical debridement of infected tissue [4]. Antibiotic therapy is often unsuccessful because developing sequestra are typically avascular [3]. Thus antibiotics and inflammatory cells cannot reach the infected area and treatment often fails. Treatment is also compromised by the emergence of antibiotic resistance [9].

*S. aureus* permanently colonizes the anterior nares of the nostrils of about 20% of the population and is transiently associated with the rest [10]. Colonisation is a risk factor for developing infection. Until recently *S. aureus* was regarded as an extracellular pathogen. However it is clear that the organism can adhere to and become internalized by a variety of host cells [6], including osteoblasts [11], and that this is likely to be important in disease pathogenesis. Uptake is promoted by fibronectin binding proteins that capture fibronectin and use it as a bridge between bacteria and the α5β1 integrin [12]. Integrin clustering results in signalling that lead to bacterial uptake into phagocytic vesicles. Once internalized bacteria can escape the phagosome and cause necrosis [13]. Slow growing variants (called small colony variants) often emerge allowing the bacteria and the infection to persist [14].

Protein A (SpA) is an important virulence factor of *S. aureus*. It binds to a variety of ligands including the Fc region of IgG [15], von Willebrand factor [16], tumour necrosis factor receptor-1 (TNFR-1) [17], the Fab-heavy chains of the Vh3 subclass [18] and the epidermal growth factor receptor (EGFR)[19]. Five ligand binding domains lie at the N-terminus followed by regions Xc and Xr that allow the protein to span the cell wall. It is covalently attached to peptidoglycan following sortase cleavage of the LPXTG motif at the C-terminus [20]. Each binding domain comprises a triple helical bundle [21]. The IgG Fc region binds to residues exposed on the face formed by helices I and II. TNFR-1 also binds to this face but there are some differences in the residues of SpA that are involved. In particular, leucine 17 is crucial for binding to IgG but not for TNFR-1 binding [17].

Although current epidemiological data suggest that *S. aureus* is responsible for greater than 80% of osteomyelitis cases there is a distinct paucity of information surrounding the mechanisms that *S. aureus* uses to weaken and trigger bone destruction. While previous studies have clearly identified mechanisms through which *S. aureus* can become internalised into osteoblasts, thus evading the immune system and avoiding antibiotic attack, these studies failed to identify mechanisms that lead to bone destruction. This study was undertaken to investigate the mechanism though which *S. aureus* binds to and triggers localised bone destruction. Understanding these mechanisms may leads to the development of new and improved therapeutic agents to treat this disease.

## Results

### *S. aureus* binding to osteoblasts is mediated by SpA

**Figure 1:**
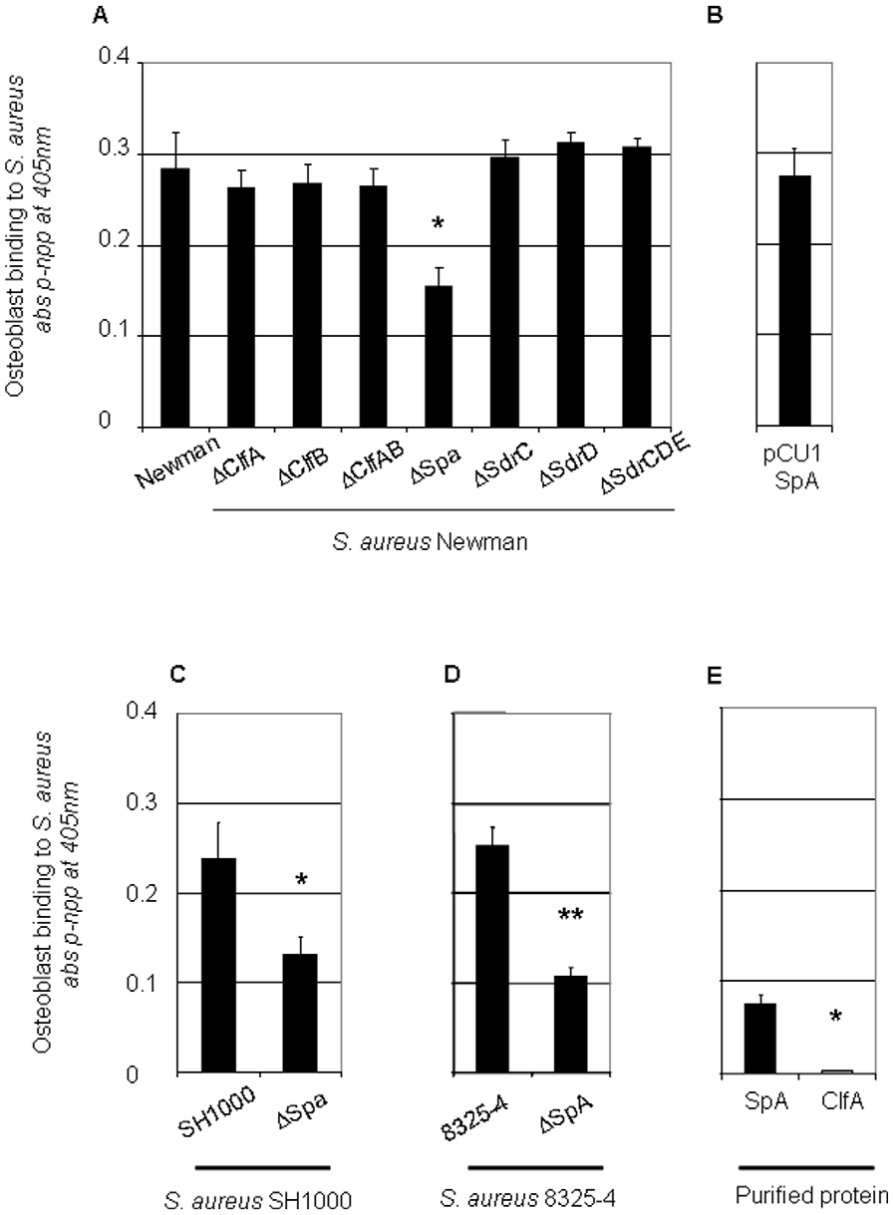
Protein A is involved in binding of *Staphylococcus aureus* to osteoblasts. Osteoblasts were allowed adhere to immobilised wildtype *S. aureus* and isogenic mutants (1×10^9^ cells/ml), (**A+B**) strain Newman, (**C**) strain SH1000, (**D**) strain 8325-4 or (**E**) purified protein A or recombinant ClfA(50 µg/ml) for 45 mins at 37°C. Adhesion was determined by measuring the intracellular enzyme alkaline phosphatase content at 405 nm in a microplate reader. *P<0.05, **P<0.01, n = 7.

The contribution of cell surface molecules to the ability of *S.aureus* to support adherence to osteoblasts was investigated using isogenic mutants. It should be noted that strain Newman does not express fibronectin binding proteins so these strains are unlikely to be internalized rapidly. Mutants defective in several surface proteins previously shown to be involved in host recognition were immobilized and the ability of osteoblasts to adhere to the bacteria was measured. Mutants defective in expression of clumping factors A (ClfA) and B (ClfB), and serine asparate repeat proteins SdrC, SdrD and SdrE expression had no effect on osteoblast binding (Fig 1A, P = NS) whereas a mutant defective in SpA bound significantly less than the wildtype (Fig 1A, P<0.05). In order to confirm that SpA was involved in binding osteoblasts, a mutant of *S. aureus* Newman was complemented with a multicopy plasmid carrying the *spa* gene, pCU1*spa*. After growth, the expression of protein A was detected by western immunoblotting (data not shown). Complementation of the *S. aureus* SpA mutant with pCU1*spa* resulted in attachment to osteoblasts to a similar extent of that of the wildtype *S. aureus* Newman (Fig 1B, P = NS to the wildtype Newman control).

In order to determine if other strains of *S. aureus,* and in particular those expressing FnBPs, could bind osteoblasts and to examine the role of SpA, strains SH1000 and 8325-4 along with their isogenic *spa*-deficient mutants were tested. In both cases a significant reduction in osteoblast binding was observed (Fig 1C, P<0.01 and Fig 1D, P<0.005, respectively). Finally, to confirm the ability of SpA to interact with osteoblasts, the purified protein was immobilized and was shown to support binding of the osteoblasts (Fig 1E, P<0.01). As a control the recombinant A domain of clumping factor A did not promote attachment.

### *S. aureus* binds to osteoblast Tumour Necrosis Factor Receptor 1

**Figure 2:**
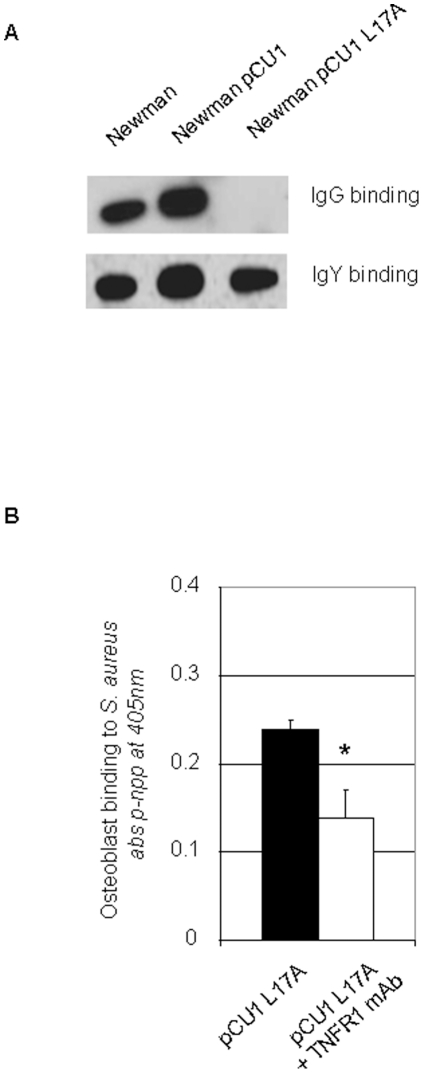
*Staphylococcus aureus* SpA binds to osteoblast tumour necrosis factor receptor 1. (**A**) *S. aureus* Newman, Newman pCU1*spa* and Newman pCU1*spa* L17A were lysed, separated on 7.5% SDS-PAGE gels, electroblotted onto PDVF membranes. Membranes were probed with either a nonspecific IgG or anti-SpA IgY antibody. Protein bands were detected using species specific horseradish peroxidase-conjugated secondary antibody and chemiluminescence. (**B**) Osteoblasts (5×10^5^ cells/ml) were preincubated with either isotype control antibody or inhibitory monoclonal antibody against TNFR-1 for 15 mins prior to addition to *S. aureus* Newman pCU1*spa* L17A. Adhesion was determined by measuring the intracellular enzyme alkaline phosphatase content at 405 nm in a microplate reader. *P<0.05, n = 7.

SpA is a multifunctional protein that has been shown to bind a number of ligands including IgG and Tumour Necrosis Factor Receptor1 (TNFR-1). Osteoblasts express high levels of TNFR-1. We investigated if SpA expressed by *S. aureus* could support binding to osteoblasts via TNFR-1. We used a strain of *S. aureus* Newman that expressed a variant of SpA where leucine 17 was substituted with an alanine in each of the five ligand binding domains. This substitution completely abolished IgG binding, but did not affect binding to TNFR-1 (Gomez et al., 2006). Immunoblotting was performed to show that the SpA variant L17A did not bind IgG. In contrast, Newman pCU1*spa* reacted with IgG in the blot. In control blots anti-SpA IgY bound to SpA from wildtype Newman, Newman pCU1*spa* and Newman pCU1*spa* L17A (Fig 2A). *S. aureus* Newman pCU1*spa* promoted adherence of osteoblasts equally as well as the Newman expressing wildtype SpA (99±1% of wildtype control, P = NS, n = 5). Furthermore, osteoblast-binding to Newman pCU1*spa* was significantly reduced by pretreatment of osteoblasts with a monoclonal anti-TNFR-1 IgG antibody (Fig 2B, P<0.05). These results indicate that immobilized *S. aureus* cells can support binding of cultured osteoblasts mediated by the interaction between SpA and TNFR-1.

### *S. aureus* SpA inhibits proliferation of osteoblasts

**Figure 3:**
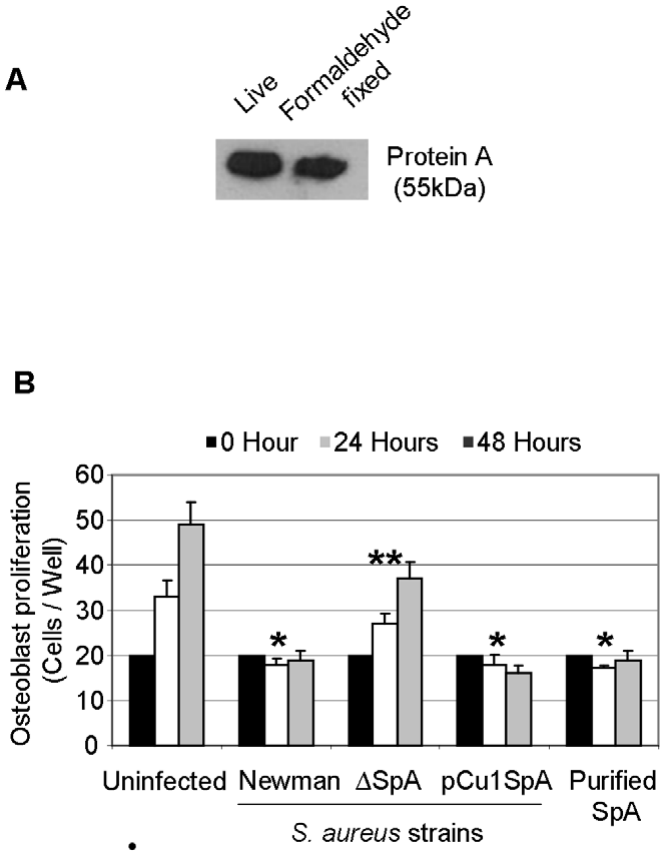
*Staphylococcus aureus* lacking SpA does not inhibit osteoblast proliferation. (**A**) Live or formaldehyde fixed *S. aureus* Newman was lysed in RIPA buffer containing 1 X protease inhibitor cocktail on ice for 10 minutes and probed with anti-protein A IgY antibodies. Protein bands were detected using species specific horseradish peroxidase-conjugated secondary antibody and chemiluminescence. (**B**) Osteoblasts (5×10^5^ cells/ml) were preincubated with either control buffer or formaldehyde fixed *S. aureus* Newman, Newman SpA deficient or Newman pCU1*spa* (1×10^9^ cells/ml) or purified protein A (50 µg/ml) for a total period of 21 days. After days 7, 14 and 21 osteoblasts were removed by trypsinization and proliferation was determined by counting cells on a haemocytometer. *P<0.01, **P = NS, n = 5.

Given the observation that *S. aureus* SpA binds to osteoblasts we next investigated if SpA had any effect on osteoblast survival or proliferation. Live *S. aureus* can use nutrients in tissue culture media to divide. This process will starve osteoblasts of essential nutrients necessary for their growth. To address this we fixed *S. aureus* in a mild formaldehyde solution aimed to maintain bacterial cell integrity yet stunt their growth. This fixation step failed to have any effect on the SpA protein as determined by western blot (Fig 3A). Formaldehyde-fixed *S. aureus* Newman was added to osteoblasts and proliferation determined after 24 and 48 hrs. In the absence of *S. aureus,* osteoblasts proliferated as expected. Addition of *S. aureus* Newman *Spa*+ ablated proliferation (Fig 3B, P<0.01). A mutant defective in SpA did not prevent proliferation (P = NS to uninfected osteoblasts). Complementation of the *S. aureus* mutant with pCU1*spa* restored the inhibitory effect to the same level as wild type (P<0,01). Finally, addition of purified protein A to osteoblasts also prevented proliferation (P<0.01). These results suggest that *S. aureus* SpA binds to osteoblasts and triggers a signal transduction pathway that inhibits osteoblast proliferation.

### *S. aureus* induces osteoblast apoptosis

**Figure 4:**
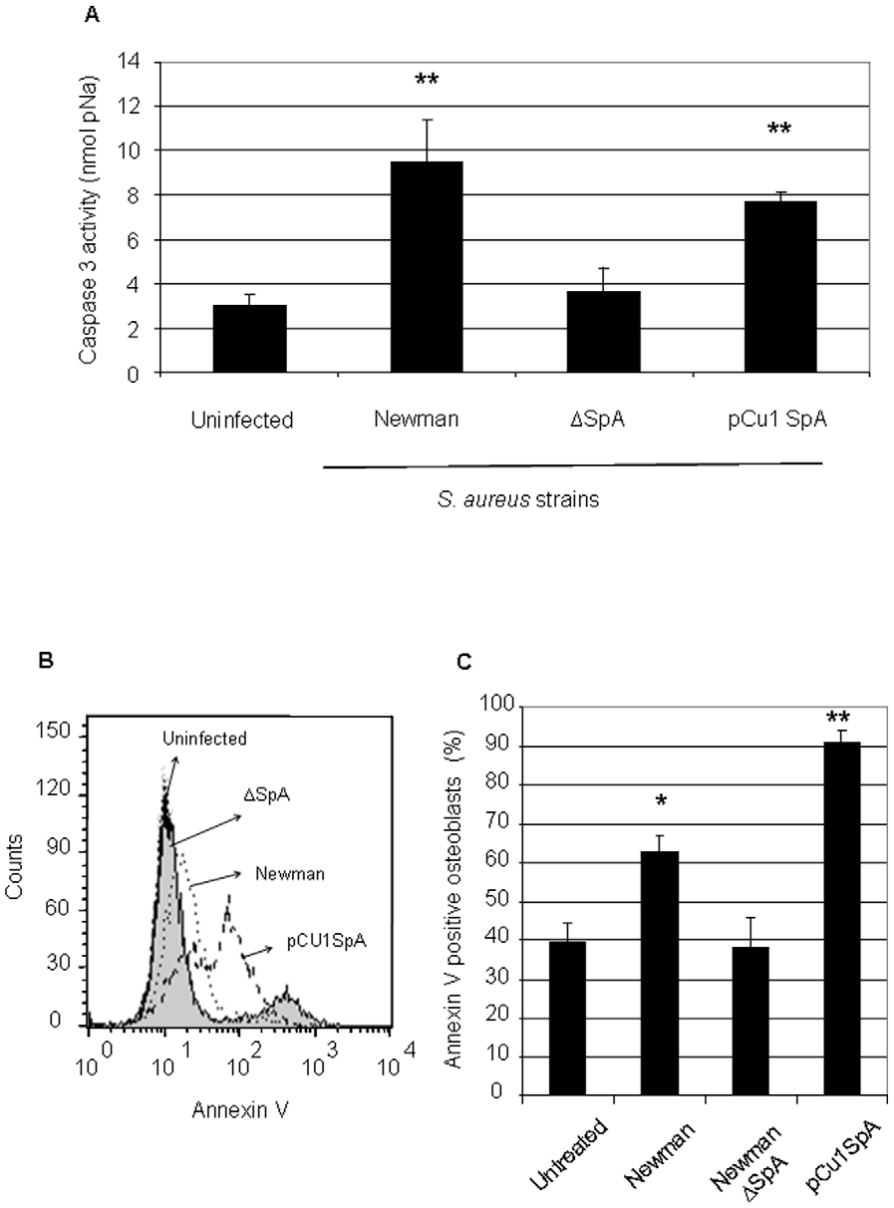
*Staphylococcus aureus* lacking SpA does not trigger osteoblast apoptosis. (**A**) Osteoblasts (5×10^5^ cells/ml) were preincubated with either control buffer or formaldehyde fixed *S. aureus* Newman (1×10^9^ cells/ml) for 24 hrs. Osteoblasts are removed, lysed and incubated with caspase 3 substrate (DEVD-pNA) for 1 hr at 37°C. Caspase 3 cleavage was measured at 405 nm in a microplate reader. (**B+C**) To measure Annexin V binding pelleted osteoblasts were re-suspended in 100 µl of FITC-Annexin V antibody. Suspensions were incubated in the dark for 15 min at RT and analysed by flow cytometry. *P<0.01, **P<0.05, n = 5.

It has been reported in the literature that *S. aureus* can induce apoptosis in osteoblasts however the mechanism was not elucidated [22], [23]. We next investigated if *S. aureus* SpA binding to osteoblasts is responsible for inducing apoptosis. Apoptosis was first determined by measuring the amount of caspase 3 cleavage following incubation of cultured osteoblasts with formalin-treated *S. aureus* cells after a 24 hour period. Caspase 3 is a downstream effector caspase important in death receptor apoptotic mechanisms. In the absence of *S. aureus*, caspase 3 cleavage was minimal. However, after incubation with wildtype *S. aureus* Newman, caspase 3 activity was significantly increased, indicating that the osteoblasts were undergoing apoptosis (Fig 4A, P<0.01). No increase in caspase-3 cleavage occurred after addition of the *S. aureus* mutant defective in SpA (P = NS compared to the uninfected osteoblasts). Complementation with pCU1*spa* induced caspase 3 activity (P<0.01). Finally, addition of purified SpA to osteoblasts also increased caspase 3 activity (data not shown, P<0.05, n = 5).

Apoptosis was also detected by measuring the amount of FITC-Annexin V binding following incubation of cultured osteoblasts with formalin-treated *S. aureus* cells after a 24 hour period. In the absence of *S. aureus* 40% of osteoblasts appeared to bind Annexin V. Addition of *S. aureus* Newman to the osteoblasts led to a significant increase in Annexin V binding (Fig 4B+C, P<0.05). In contrast, addition of the *S. aureus* mutant defective in SpA failed to induce apoptosis above the uninfected levels (P = NS). Addition of *S. aureus* pCU1*spa* to the osteoblasts induced significant apoptosis (P<0.001). *S. aureus* pCU1*spa* induced significantly more apoptosis than the wildtype *S. aureus* strain (P<0.005).

### *S. aureus* inhibits mineralisation of osteoblasts

**Figure 5:**
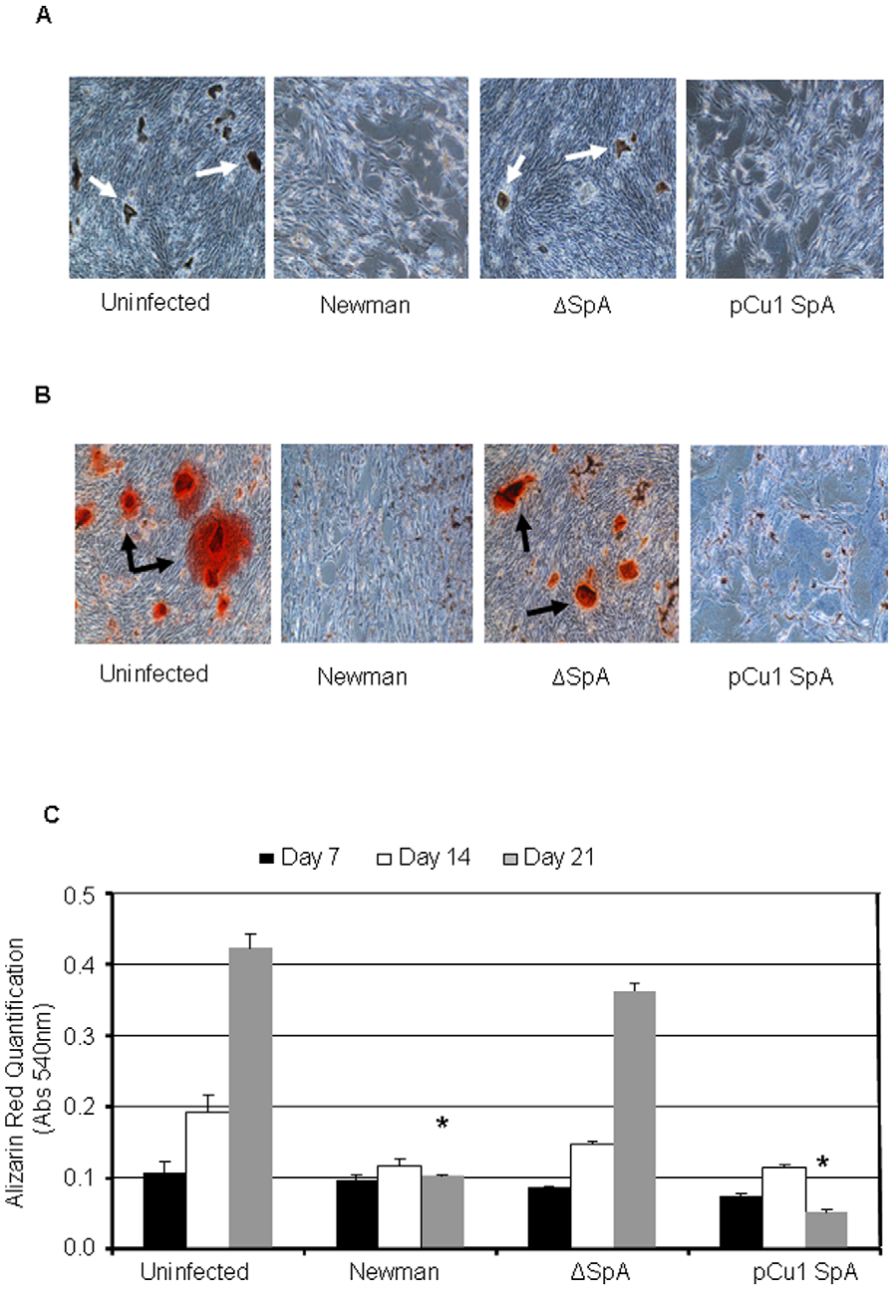
*Staphylococcus aureus* induces RANKL and inhibits OPG in osteblasts. *S. aureus* (1×10^9^ cells/ml) was added to the osteoblasts for 24 hrs. Two hundred micro litres of the RIPA buffer was added to each well resulting protein was removed and centrifuged at ×10,000 g for 2 minutes. (A) RANKL or (B) OPG was detected using an ELISA kit. *P<0.001, **P<0.0001, n = 3.

Following matrix deposition, osteoblasts facilitate the calcification and mineralisation of bone matrix thereby ensuring strength and rigidity to the skeletal system. We next investigated the effect of *S. aureus* on the mineralisation of osteoblasts. Mineralisation typically begins between day 7–10 and can be detected by staining the phosphates and calcium-rich deposits on the osteoblasts. Representative images obtained by brightfield microscopy showing staining for phosphates (von Kossa) from day 21 is shown in Fig 5A and for calcium deposition (Alizarin red) is shown in Fig 5B. Uninfected osteoblasts showed signs of both phosphate (Fig 5A) and calcium deposition (Fig 5B). Addition of either *S. aureus* Newman or *S. aureus* complemented with pCU1*Spa* prevented both phosphate (Fig 5A) and calcium deposition (Fig 5B). Critically, osteoblasts showed signs of both phosphate (Fig 5A) and calcium deposition (Fig 5B) when *S. aureus* SpA mutant was added. Quantification of the calcium deposition (alizarin red staining) supported these results demonstrating that over a 21 day period osteoblasts from both the uninfected and *S. aureus* SpA mutant stained for calcium deposition similarly (Fig 5C, P = NS), whereas *S. aureus* Newman and *S. aureus* complemented with pCU1*Spa* failed to do so (Fig 5C, P<0.01 and P<0.001, respectively).

### *S. aureus* induced RANKL expression in osteoblasts

**Figure 6:**
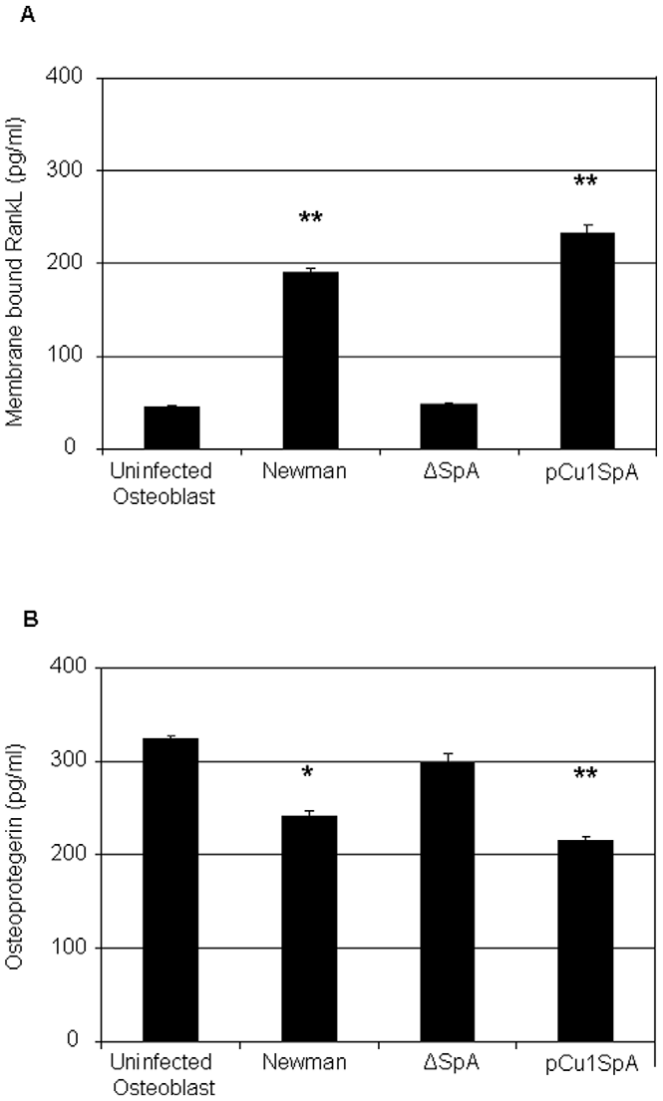
*Staphylococcus aureus* prevents osteoblast mineralisation. Osteoblasts (5×10^5^ cells/ml) were preincubated with either control buffer or formaldehyde fixed *S. aureus* Newman (1×10^9^ cells/ml) for a total period of 21 days. (**A**) Following a 21 day infection von kossa stain was added to the osteoblasts to determine phosphate deposition. Phosphate nodules are indicated by white arrows. (**B**) Following a 21 day infection alizarin red stain (2%) was added to the osteoblasts. Calcium nodules are indicated by black arrows. (**C**) Staining was quantified by leeching the cells of the dye and reading absorbance at 540 nm. Representative images from day 21 for both stains were obtained using 20x bright field microscopy.. *P = NS, **P<0.05, ***P<0.01, n = 3.

RANKL is a key molecule involved in bone remodeling. Once expressed on osteoblasts it initiates bone resorption. Previous studies have shown that RANKL is upregulated in patients with staphylococcal osteomyelitis. We therefore investigated if *S. aureus* SpA leads to RANKL expression by osteoblasts. Quantitative analysis of membrane bound RANKL expression on osteoblasts after 24 hrs demonstrated that in uninfected osteoblasts a low level of RANKL was expressed. Addition of *S. aureus* Newman significantly increased RANKL expression following 24 hrs (Fig 6A. P<0.0001). Addition of the *S. aureus* SpA mutant failed to express significant levels of membrane bound RANKL compared to the uninfected sample (P = NS). However, complementation of the *S. aureus* mutant with pCU1*spa* led to significant expression of membrane bound RANKL (P<0.0001).

Osteoprotegerin is a natural inhibitor of RANKL. By binding RANKL, osteoprotegrin prevents transcription via NF-κB and activation of osteoclasts, thereby preventing bone resorption. When RANKL expression is high osteoprotegrin levels are low and vice versa. Therefore we investigated this inverse relationship by measuring osteoprotegrin in infected and uninfected osteoblasts. Consistent with these findings osteoprotegrin was high in uninfected osteoblasts and significantly lower in *S. aureus* infected osteoblasts following 24 hrs (Fig 6B, P<0.001). Osteoprotegrin levels were high in cells exposed to the *S. aureus* SpA mutant and low in the *S. aureus* complemented with pCU1*Spa* (P<0.001). Collectively these results suggest that SpA binding to the osteoblasts is responsible for RANKL expression leading to activation of osteoclasts and bone breakdown.

## Discussion

Bone is normally resistant to infection. However trauma, surgery to the skeleton or placement of a foreign body such as an orthopaedic implant may expose this otherwise sterile environment to infection. *S. aureus* is a common human pathogen which lives harmlessly on the skin and mucous membranes of healthy individuals. It is isolated from more than 80% of patients with bone infection or osteomyelitis [13]. Upon exposure to the bone, *S. aureus* induces a severe inflammatory response followed by progressive bone destruction and loss of the vasculature. As a result, sections of dead bone separate themselves from the healthy bone forming a sequestra [7]. This area of dead infected tissue is inaccessible to immune cells or to antibiotics, leading to persistent chronic infection. This is further complicated by the rapid emergence of strains of *S. aureus* that are resistant to multiple antibiotics which makes treatment of this disease very difficult [3]. A better understanding of the interactions between *S. aureus* and the bone may aid in the development of novel therapeutics to treat this disease.

Previous reports demonstrated that *S. aureus* is capable of invading osteoblasts from human, murine and chicken sources in both culture and in vivo [24]; [25]; [26]. Invasion is dependent on the *S. aureus* fibronectin binding proteins FnbpA and FnbpB which bind to the extracellular matrix protein fibronectin and serves as a bridging molecule to integrin α5β1 [12]. In the absence fibronectin or using mutants deficient in FnbpA and FnbpB, *S. aureus* invades osteoblasts very poorly. Interestingly bacteria still adhere to the osteoblast surface [11] even in the absence of FnbpA and FnbpB. Thus, *S. aureus* is also capable of directly interacting with osteoblasts in the absence of extracellular matrix proteins such as fibronectin.

*S. aureus* expresses several components that are capable of interacting with osteoblasts. A major virulence factor known to promote evasion of/or interference with the host immune system is capsular polysaccharides expressed on the bacterial cell surface [27]; [28]. Deletion of capsule from *S. aureus* Newman or using the capsule negative strain SH1000 failed to have any effect on binding, suggesting that the polymeric carbohydrates that are found in the capsule do not mediate attachment to osteoblasts (data not shown). *S. aureus* owes much of its pathogenicity to an array of cell wall-anchored surface proteins. These adhesins facilitate binding to various matrix proteins or host cells [29].

SpA is one of the most prevalent cell wall proteins on *S. aureus* and appears to play an important role in the success of *S. aureus* as a human pathogen [10]; [16]. By binding the Fc portion of immunoglobulin, SpA assists *S. aureus* in evasion of phagocytosis by neutrophils [30]. SpA also binds to von Willebrand factor which may play a role in supporting platelet adhesion in the early stages of thrombosis[16]. In addition, Gomez and colleagues demonstrated that SpA binds to the EGFR to regulate TNFR1 availability[19]. Deletion of SpA in several different strains of *S. aureus* (Newman, SH1000, 8325-4) significantly reduced binding to osteoblasts. Complementing the SpA mutant with the pCU1*spa* plasmid restored binding.

Recently, *S. aureus* SpA was shown to bind directly to TNFR-1 in lung epithelial cells resulting in proinflammatory signalling in the pathogenesis of staphylococcal pneumonia[17]. Osteoblasts also express high levels of the TNFR-1[31] and engagement with its ligand TNFα has been implicated in a wide spectrum of bone diseases including osteoporosis and rheumatoid arthritis[32]. Here we demonstrate that inhibition of TNFR-1 on osteoblasts significantly reduced binding to *S. aureus*. These results suggest that SpA maybe binding to the osteoblast TNFR-1, however additional experiments using TNFR-1 null mice or siRNA to reduce the receptor are required to confirm this.

TNFR-1 is known as a death receptor because its engagement triggers a series of events that culminates in apoptosis [33]. Several reports have demonstrated that *S. aureus* can induce apoptosis in osteoblasts [22], [23]. However the mechanism by which this occurs has not yet been elucidated. Caspase 3 is a key component of the proteolytic cascade that leads to apoptosis which is typically followed by membrane blebbing. Active caspase 3 and extrusion of annexin V (as a measure of membrane blebbing) was detected in the uninfected control osteoblasts and is most likely due to apoptosis and cell turnover in the *in vitro* osteoblast tissue culture system. Consistent with previous observations, when osteoblasts were exposed to *S. aureus*, caspase 3 and annexin V were significantly increased [23]. The SpA mutant yielded similar results to the uninfected osteoblasts. The complemented mutant or addition of purified protein A increased caspase 3 and annexin V in osteoblasts to levels similar to *S. aureus* Newman. Previous results demonstrated that *S. aureus* α-toxin induced apoptosis in Jurkat cells via the Bcl-2-controlled mitochondrial death pathway which involves caspase 3 [34] However this mechanism is not playing a role in these experiments as the *S. aureus* cells are formalin-fixed and cannot produce α-toxin. These results therefore suggest that *S. aureus* SpA binds to osteoblasts, possibly through an interaction with the death receptor TNFR-1 which induces caspase 3 activation and membrane blebbing with an end point of apoptosis. *S. aureus-*induced apoptosis is not unique to osteoblasts as endothelial and epithelial cells undergo apoptosis following *S. aureus* infection [35]–[37].

Mineralisation is a process where phosphate and calcium becomes deposited in bone[1]. This gives the bones additional strength and rigidity. During *S. aureus* infection, mineralisation (phosphate and calcium deposition) is completely inhibited. Deletion of SpA from *S. aureus* allowed both phosphate and calcium deposition. Interestingly, when TNFα binds to its receptor it results in inhibition of bone mineralisation. These results further suggest that engagement of the TNFR leads to a signal that prevents mineralisation such as the one that *S. aureus* SpA provides.

RANKL is a homotrimeric molecule displayed on the membrane of osteoblasts that stimulates differentiation in osteoclasts and is a key induction molecule involved in bone resorption leading to bone destruction[38]. Osteoprotegerin (OPG) is also produced and released by osteoblasts and is an endogenous inhibitor of RANKL signalling. It typically acts as a decoy receptor that binds to RANKL and prevents its association with RANK with the net result of preventing excessive bone destruction through activation of osteoclasts[39]. Consistent with previous results we found that *S. aureus* infection of osteoblasts led to a significant increase in RANKL expression in their membrane[40]. The increase in RANKL is likely to trigger osteoclast-induced bone resorption and bone destruction and may help explain why patients with osteomyelitis have significant bone loss. These results are consistent with the observation that TNFα binding to TNFR-1 on osteoblasts triggers ostoclast differentiation and subsequent bone destruction[41]. Critically, deletion of SpA from *S. aureus* prevented RANKL expression, possibly because *S. aureus* is unable to bind to TNFR-1 to trigger an increase in RANKL expression.

**Figure 7:**
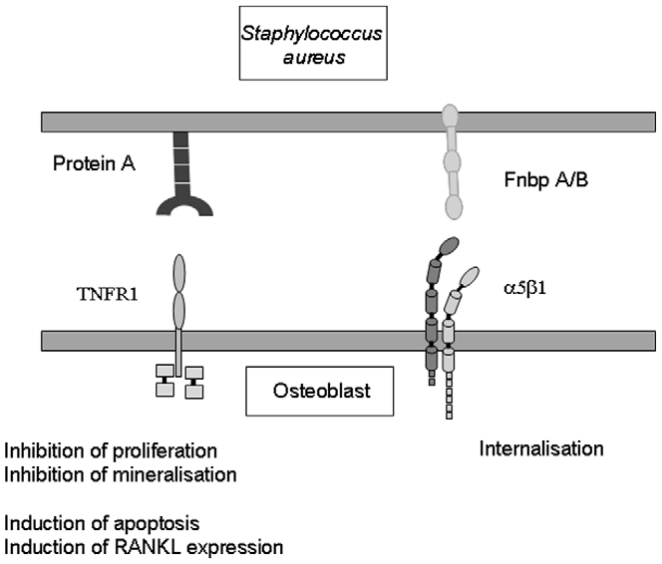
Proposed model of *Staphylococcus aureus* – osteoblast interaction.

To date the mechanism by which *S. aureus* causes weakening of the bones in osteomyelitis is not fully understood. Previous results demonstrate that osteoblasts internalise the *S. aureus* via an indirect interaction between the fibronectin binding proteins that bind fibronectin and form a bridge to osteoblast α5β1 as a result *S. aureus* can evade immune responses and antibiotics. Here we describe for the first time that the major *S. aureus* virulence protein, SpA also binds directly to osteoblasts. This interaction results in the generation of multiple signals leading to inhibition of osteoblast proliferation, induction of osteoblast apoptosis, inhibition of mineralisation and release of mediators capable of inducing bone resorption via osteoclast activation (Fig 7). Given the finding that inhibition of the TNFR1 significantly reduces binding to osteoblasts it is tempting to suggest that this receptor plays a role in the pathogenesis of the disease process however, additional studies are required to confirm the role of the TNFR-1 in mediating these responses. Regardless of this, SpA binding to osteoblasts is an important step as it occurs in the absence of matrix proteins and is most likely the initial event in the development of bone infection. Results presented in this study provides evidence for the first time that SpA is likely to play a critical role in the success of *S. aureus* as a human pathogen in osteomyelitis and is a potential novel drug target for the treatment of this debilitating disease.

## Methods

### Bacterial strains and growth conditions

The *Staphylococcus aureus* strains used in this study are listed in Table 1. They were grown to stationary phase at 37°C in Brain Heart Infusion broth (Oxoid, Basingstoke, United Kingdom). Bacteria were harvested and washed by centrifugation at 15,000 g for 5 min and finally re-suspended in phosphate buffered saline (PBS) pH 7.4. For all experiments, *S. aureus* suspensions were adjusted to 1×10^9^ cells/ml. In some studies *S. aureus* were treated with 100 µg/ml trypsin-EDTA for 30 minutes with constant rotation, in order to remove cell wall proteins. Following this, samples were centrifuged at 15,000 g for 5 min, then the supernatant was removed and the pellet was re-suspended in 1 ml PBS. Trypsin-treated *S. aureus* were adjusted to 1×10^9^ cells/ml for adhesion studies.

**Table 1:** List of strains of *Staphylococcus aureus* used in this study.

Strain or plasmid	Relevant Characteristics	Reference
***S. aureus***		
Newman	NCTC 8178 wildtype	Duthie and Lorenz, 1952
Newman *clfA*	*clfA*:: Em^r^ defective in clumping factor A	McDevitt et al 1994.
Newman *clfB*	Newman *clfB:: lacZ* Em^r^ defective in clumping factor B	McAleese et al, 2001
Newman *spa*	Newman *spa*:: Ka^r^ defective in protein A	O'Brien et al, 2002
Newman *sdrC*	*sdrC*:: pG^+^Host Em^r^ defective in Serine a*spa*rtate repeat protein C	O'Brien et al, 2002
Newman *sdrD*	*sdrD*:: pG^+^Host Em^r^ defective in Serine a*spa*rtate repeat protein D	O'Brien et al, 2002
Newman Δ*sdrCDE*	Δ*sdrCDE*:: Tc^r^. Deletion mutant lacking SdrC SdrD and SdrE	O'Brien et al, 2002
Newman *cap*	*cap*:: Tc^r^ defective in capsular polysacccharide	Wann et al, 1999
SH1000	8325-4 with repaired defect in *rbsU*	Horsburgh et al 2002
SH1000 *spa*	*spa*:: Tc^r^ transduced from 8325-4 *spa*	This study
8325-4	NCTC8325 cured of prophages	Novick 1967
8325-4 *spa*	*spa*:: Tc^r^. Protein a defective mutant	Hartlieb et al, 2000
Newman *clfA clfB*	*clfA*:: Em^r^ *clfB*: Tc^r^ Defective in clumping factors A and B	Ni Eidhin et al, 1998
***Plasmids***		
pCU1*spa*	Shuttle vector capable of replicating in *E. coli* and *S. aureus*. Cm^r^ Ap^r^ *spa* gene cloned into pCU1	This study
pCU1 *spa*L17A	Expresses *spa* with an L17A substitution in each IgG binding domain	This study

### Bacterial strains and plasmids

*S. aureus* wildtype strains and mutants are listed in Table 1. pCU1*spa* was constructed by amplifying DNA encoding the *spa* gene including the promoter region and cloning into the multiple cloning site. The region encoding the SpA IgG binding domains EDABC was deleted by inverse PCR and an *Acc*I site introduced forming pCU1Δ*spaΔEDABC*. The DNA region encoding the SpA domains bearing an L17A substitution in each ligand binding domain and flanked by *Acc*I sites was synthesized commercially. It was cut with *Acc*I and cloned into the introduced *Acc*I site in pCU1Δ*spaΔEDABC*. The resulting plasmid, pCU1*spa*L17A. was verified by DNA sequencing.

### Cell culture conditions

The mouse clonal MC3T3-E1 pre-osteoblastic cell line (ATCC, Middlesex, UK) was used for these experiments. This is a standard osteoblast cell line and is used routinely for the assessment of osteoblasts in different culture conditions [42], [43], [44] These cells were cultured in standard tissue culture flasks containing α-MEM supplemented with 10% FBS, 2% penicillin-streptomycin solution and 1% L-glutamine (Biosera Ltd., East Sussex, United Kingdom). The media was replaced every 3–4 days and after confluency, cells were harvested using trypsin and re-suspended in medium.

### Osteoblast proliferation assay

Overnight cultures of *S. auerus* were harvested, washed and fixed in 4.8% formaldehyde under rotation and washed to eliminate any residual formaldehyde. *S. aureus* were centrifuged at 15,000 g for 5 minutes were re-suspended in α-MEM. A 6-well plate was coated with 1 ml formaldehyde fixed *S. aureus* (1×10^9^ cell/ml) or 50 µg/ml of purified protein A. The plate was incubated at 37°C with 5% CO_2_ for 2hrs. Following this, the unbound bacteria or purified protein A was removed by gentle aspiration. MC3T3-E1 osteoblasts (5×10^5^ cells/ml) were added into each well. Un-infected osteoblasts were seeded and cultured in the absence of bacteria as a control. Osteoblast proliferation was determined by counting cells on a haemocytometer in a 1∶1 dilution with Trypan Blue.

### Osteoblast binding studies

Microtitre plates (96 well) were coated with 100 µl of bacteria (1×10^9^ cells/ml) or purified protein A (50 µg/ml) (Sigma, Wicklow, Ireland). The plate was incubated at 37°C for 2 hours. Following this, the plate was washed with PBS and blocked with 1% bovine serum albumin (BSA) for a further 1 hour at 37°C. The plate was then washed once in PBS to remove any unbound bacteria or protein. MC3T3-E1 cells (1.5×10^6^ cells/ml) were added to each well and allowed to adhere for 45 minutes at 37°C. In some studies, osteoblasts were pre-incubated with 50 µg/ml of anti-TNFR-1 antibody or isotype control antibody (Santa Cruz, Heidelberg, Germany) for 30 minutes at room temperature (RT) prior to their addition to the plate. Each well was gently washed with 100 µl PBS to remove any non-adhered osteoblasts. Adherent osteoblasts were then lysed with 100 µl lysis buffer containing a substrate for acid phosphatase (0.1 M Na acetate pH 5.5, 0.1% Triton X-100, 10 mM p-nitrophenol phosphate) and incubated for 20 minutes at 37°C and the A_405nm_ read in a microplate reader (Wallac Victor2, Perkin Elmer, Cambridge, United Kingdom).

### Osteoblast apoptosis

Caspase 3 is a downstream effector caspase important in death receptor apoptotic mechanisms. Caspase 3 activity was measured using a Caspase 3 colorimetric assay kit (Clontech Laboratories, CA (US) according to manufacturer's instructions. Briefly, cultured MC3T3-E1 osteoblasts were incubated with *S. aureus* or purified protein A (50 µg/ml). Untreated osteoblasts were used as a negative control and 100 µM dexamethasone- treated osteoblasts as a positive control. Cell lysates were incubated with the Caspase 3 substrate DEVD-pNA at 37°C for 1 hour. Samples were read at A _405nm_ in a microplate reader (Wallac Victor2, Perkin Elmer, Cambridge, United Kingdom).

Annexin V-FITC staining to infected osteoblasts was carried out according to the manufacturer guidelines (Trevigen). Cultured MC3T3-E1 osteoblasts were infected over 24 h with 4.8% formaldehyde fixed *S. aureus* Newman WT, NewmanΔ*Spa* and Newman pCu1*Spa* (OD = 1.6), un-infected osteoblasts were used as negative control. Briefly un-infected and infected cells (1.5×10^5^) were collected by centrifugation at 300×g for 5 min at RT. Pellets were washed once in cold (4°C) PBS and re-centrifuged at 300 ×g for 5 min at RT. Pellets were then re-suspended in 100 µl of Annexin V Incubation Reagent. Suspensions were incubated in the dark for 15 min at RT. Following addition of 400 µl of 1 X Binding Buffer, samples were processed by flow cytometry.

### Immunoblotting

*S. aureus* Newman (formaldehyde fixed or unfixed) were lysed in RIPA buffer containing 1X protease inhibitor cocktail on ice for 10 minutes. Lysates were separated on a 10% sodium dodecylsulfate polyacrylamide (SDS-PAGE) gel. Proteins were electroblotted onto polyvinyldifluoride membranes (Roche, UK) for 1 hr. The membrane were probed with either a primary IgG antibody (clone 3E8) or primary chicken anti-protein A IgY antibody (clone *SPA*-27) overnight at 4°C with constant inversion. Unbound antibody was removed by 3×10 min washes with TS buffer. Protein bands were detected using species-specific horseradish peroxidase-conjugated secondary antibody and developed by chemiluminescence.

### Quantification of RANKL and OPG

MC3T3-E1 cells (1×10^6^ cells/well) were seeded on six well plates. *S. aureus* was added to the osteoblasts for 24 hours. Two hundred microlitres of the RIPA buffer was added to each well resulting protein was removed and centrifuged at ×10,000 g for 2 minutes. RANKL or OPG was detected using an ELISA kit (R&D Systems, Minneapolis, MN) according to manufacturer's instructions.

### Statistical Analysis

Statistics were performed using SSC-Stat V2.12. Data shown are the means plus or minus standard error of the mean (SEM). Comparisons between mean values were performed using a 2-tailed student T-test.
